# What molecular imaging of cancer patients can teach us about COVID-19

**DOI:** 10.1140/epjp/s13360-022-03262-w

**Published:** 2022-09-19

**Authors:** Silvana Del Vecchio, Cristina Terlizzi, Sara Pellegrino, Giovanna G. Altobelli, Rosa Fonti

**Affiliations:** Department of Advanced Biomedical Sciences, University of Naples “Federico II”, Naples, Italy

## Abstract

COVID-19 pandemic had a great impact on health systems and cancer care worldwide. Patients with cancer who develop COVID-19 are at high risk of severe outcomes and clarifying the determinants of such vulnerability of cancer patients would be of great clinical benefit. While the mechanisms of SARS-CoV-2 infection have been elucidated, the pathogenetic pathways leading to severe manifestations of the disease are largely unknown. Critical manifestations of COVID-19 mainly occur in elderly patients and in patients with serious comorbidities including cancer. Efforts to understand the intersection of pathways between severe manifestations of COVID-19 and cancer may shed light on the pathogenesis of critical illness in COVID-19 patients. Here, we will focus our attention on two major fields of potential intersection between COVID-19 and cancer, namely the dysfunction of immune system and the prothrombotic state that can occur in both COVID-19 and cancer patients, testing whether cancer imaging can provide clues to better understand such interactions.

**Graphical abstract:**

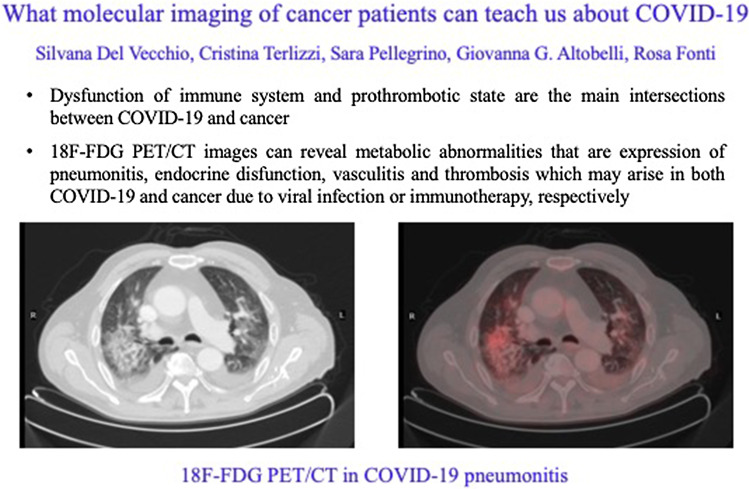

## Introduction

The COVID-19 pandemic caused by the rapid transmission of SARS-CoV-2 infection had an extraordinary impact on health systems and dramatic socio-economic consequences worldwide. The introduction of vaccines and vaccination campaign mitigated in part the pandemic effects but the emergence of viral variants is still a concern. Critical manifestations of COVID-19 mainly occur in elderly patients and in patients with serious comorbidities. In particular, severe outcomes of COVID-19 have been observed in patients with cancer especially those with hematological malignancies [1]. While the molecular mechanisms of SARS-CoV-2 infection have been elucidated [2], the pathogenetic pathways leading to severe manifestations of the disease are largely unknown. Efforts to understand the intersection of pathways between severe manifestations of COVID-19 and cancer may shed light on the pathogenesis of critical illness in COVID-19 patients.

SARS-CoV-2 enters host cells through binding of spike (S) protein, a structural protein of the virus, to angiotensin-converting enzyme 2 (ACE2) on the surface of target cells and employs the cellular serine protease TMPRSS2 for S protein priming [2]. Cleavage of the S protein is followed by membrane fusion and viral internalization along with ACE2 by endocytosis. After infection, some patients will show mild flu-like symptoms whereas in other patients a complex and not completely elucidated pathogenic cascade, involving a cytokine storm, will lead to a severe pneumonia, rapidly evolving to acute respiratory distress syndrome (ARDS), and to multiorgan damage [3]. Furthermore clinical observations and autopsy findings indicate that COVID-19 patients develop a generalized thrombogenic vasculopathy with arterial, venous and microvascular thrombosis [4] that can cause multiorgan damage in critical illness.

Based on these observations we will focus our attention on two major fields of potential intersection between COVID-19 and cancer, namely the dysfunction of immune system and the prothrombotic state that can occur in both COVID-19 and cancer patients, testing whether cancer imaging can provide clues to better understand such interactions.

Molecular imaging with 18F-Fluorodeoxyglucose (18F-FDG) PET/CT is the current imaging modality for staging of a variety of malignant tumors and for evaluation of tumor response to treatment with a great impact on the management of cancer patients. The widespread use of 18F-FDG relies upon the high 18F-FDG avidity of cancer cells due to their dependence from glucose supply for energy production through aerobic glycolysis. On the other hand, 18F-FDG PET/CT has been exploited also for detection of inflammatory and infection diseases since immune cell activation initiates a program of enhanced proliferation and differentiation that is supported by an increased glucose consumption through aerobic glycolysis.

## SARS-CoV-2 and cancer immune system

The severe manifestations of COVID-19 have been mainly ascribed to a cytokine storm, a fast developing severe condition characterized by a hyperinflammatory state with persistently high serum levels of several cytokines and excessive activation of immune cells that can lead to ARDS, disseminated vascular coagulation, remote organ failure and death [5].

Immune system functions of cancer patients are generally suppressed as a consequence of the intrinsic ability of cancer cells to evade immune system and the immunosuppressive therapeutic regimens administered to cancer patients. However, the recent adoption of immunotherapy in many types of tumor, by reverting the mechanisms of cancer evasion, can overactivate immune system in cancer patients causing immune-related adverse events that are similar to clinical manifestations of SARS-CoV-2 patients. This suggests that immune-related adverse events in cancer patients and severe clinical manifestations in SARS-CoV-2 patients may share common mediators and pathogenic pathways.

A life-threatening immune-related adverse effect is the cytokine release syndrome (CRS) that is more frequent in patients receiving T-cell engaging immunotherapeutic agents. This syndrome is characterized by a variety of symptoms ranging from flu-like symptoms such as cough and tachypnea to severe manifestations like ARDS, vascular leakage, disseminated intravascular coagulation, and multiorgan system failure [6]. The pathogenesis of CRS remains largely unknown. However, it has been proposed that binding of bispecific antibodies or CAR-T cell receptor to cognate antigen is followed by activation of bystander immune cells and endothelial cells. This activation may result in a massive release of cytokines including IL-6, IFNγ, IL-10, IL-2 and TNFα that in turn will cause the most detrimental effects in patients if not counter-balanced by homeostatic mechanisms. Due to the clinical similarity with cytokine storm of COVID-19 patients, it would be important to know whether the systemic cytokine profiles of patients who develop CRS in response to T-cell engaging immunotherapy are similar to those found in COVID-19 patients.

In a recent systematic review and meta-analysis [7] including 25 COVID-19 studies and 1245 patients, among whom 650 with severe and 367 with critical disease, the authors analyzed the serum level of IL-6 and other inflammatory cytokines. They found that although increased, IL-6 levels in severe and critical COVID-19 patients were 100 times significantly lower than in patients with CAR-T cell-induced CRS (*n* = 72 with CRS grade ≥ 3); 27 times significantly lower than in patients with sepsis (*n* = 5320) and 12 times significantly lower than in patients with ARDS unrelated to COVID-19 (*n* = 2767). Furthermore, IL-8 levels in COVID-19 patients were lower than in patients with sepsis, hyperinflammatory ARDS and CAR-T cell-induced CRS, whereas they were similar to those found in patients with hypoinflammatory ARDS. Moreover, IFNγ was not increased in patients with COVID-19 whereas TNFα levels were lower in COVID-19 patients than in patients with sepsis and CAR-T cell-induced CRS. Although the cytokine profile can greatly vary in different pathological conditions, these observations suggest that the systemic cytokine profile of COVID-19 patients does not overlap with that of other non-COVID-19 comparable disorders. Therefore, these findings indicate that the pathogenesis of multiorgan damage in COVID-19 may have different molecular basis, namely: (a) involvement of different cytokines; (b) a higher sensitivity of COVID-19 patients to the detrimental effects of inflammatory cytokines; (c) direct or indirect viral injury; (d) endovasculitis and thrombosis; (e) dysregulation of renin angiotensin system.

In addition to CRS mainly associated to T-cell engaging therapy, a large spectrum of immune-related adverse effects is often observed in cancer patients treated with immune checkpoint inhibitors. The administration of CTLA-4, PD-1 or PDL-1 inhibitors, by overactivating immune system, can result in the inflammation of virtually any organ. Gastrointestinal tract, endocrine glands, skin and liver are often involved by drug-induced inflammation and less frequently central nervous system, cardiovascular, pulmonary, musculoskeletal and hematological systems can be affected by these adverse events. Therefore, cancer patients during treatment with immune checkpoint inhibitors can show colitis, hepatitis, myocarditis, hypophysitis, pneumonitis, vasculitis, peripheral neuropathies, autoimmune haemolitic anemia and other adverse events. Many of these disorders are also observed in COVID-19 patients including pneumonitis, myocarditis, gastrointestinal symptoms, endocrine disfunctions, hepatitis, acute kidney injury, neurological symptoms and other manifestations of multiorgan damage. In pneumonitis, although the CT imaging pattern of COVID-19 is quite variable depending on the severity of the disease, the most frequently observed pattern is characterized by peripheral, bilateral or multifocal ground glass opacities with or without parenchymal consolidations usually distributed at the periphery and in lower lung lobes [8]. This pattern is also found in immune-related pneumonitis and differential diagnosis between the two diseases is difficult when based only on CT images. In both conditions, 18F-FDG uptake is increased in lung abnormalities and does not help in differential diagnosis. In addition to lung lesions, 18F-FDG uptake can be observed in lymph nodes, blood vessels, bone marrow, spleen and less frequently in thyroid and salivary glands [9]. However, PET imaging findings of COVID-19 are not so well encoded as for CT lung abnormalities since 18F-FDG PET/CT is not usually performed during the acute phase of the disease and 18F-FDG findings mainly refer to patients with COVID-19 sequelae. Nevertheless, 18F-FDG PET/CT images can reveal metabolic abnormalities that are expression of endocrine disfunction, vasculitis and thrombosis in COVID-19 patients.

Although the mechanisms of immune-related adverse events of checkpoint inhibitors are not completely elucidated, it is conceivable that the similar pattern of multiorgan damage found in COVID-19 patients may result from common pathogenetic mechanisms including T-cell activation with consequent T-cell proliferation and multiorgan infiltration, reduction of Treg function and survival, production of cytokines such as IL-17A, TNF, IFNγ and IL-2 [10].

## Prothrombotic state of SARS-CoV-2 and cancer patients

**Fig. 1:**
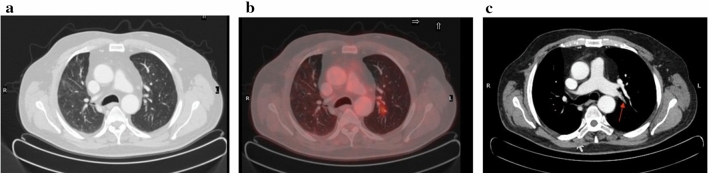
18F-FDG PET/CT performed in a 64-year-old patient two months after the acute phase of COVID-19: transaxial images of contrast-enhanced CT of the thorax with pulmonary (**a**) or mediastinal (**c**) window and corresponding PET/CT fusion images (**b**). Focal FDG uptake was observed in the left paramediastinal region (**b**) corresponding to an endoluminal ipodensity in the superior branch of the left pulmonary artery (**c**, arrow)

The other major field of potential intersection between COVID-19 and cancer is the prothrombotic state present in both diseases. A number of studies reported the association between cancer and thrombosis with approximately four–sevenfold increased risk of venous thromboembolism for cancer patients as compared to general population [11]. Pancreatic and brain cancer patients have a higher risk of venous thromboembolism than breast and prostate cancer patients. Moreover, patients with metastatic disease have a higher risk than those with localized tumors. Many factors including tissue factor expression, circulating microparticles, inflammatory cytokines such as TNFα and IL-1, high levels of plasminogen activator inhibitor, growth factors including VEGF, G-CSF and FGF, secretion of platelet aggregation agonists and neutrophil extracellular traps (NETs) were involved in the prothrombotic state associated with cancers [12]. Similarly in COVID-19 patients, the prothrombotic state is confirmed by increased levels of D-dimer, fibrinogen, factor VIII (FVIII), von Willebrand factor (vWF) and decreased antithrombin [13]. Although angioCT is the method of choice in patients with suspected pulmonary thromboembolism, 18F-FDG PET/CT was able to detect unsuspected pulmonary thrombi in COVID-19 patients (Fig. 1).

The mechanisms underlying the occurrence of thromboembolism in COVID-19 patients are largely unknown but they involve release of cytokines such as IL-6, IL-7 and TNF, endothelial injury, activation of monocytes and macrophages, complement-mediated microangiopathy, dysregulation of renin-angiotensin system and NETs. A growing body of evidence has recently highlighted the potential pathogenetic role of NETs in the multiorgan damage of COVID-19 patients.

NETs are web-like structures that are released by activated neutrophils in the extracellular environment to entrap and kill different pathogens. They are composed by ds-DNA, citrullinated hystones, myeloperoxidase, neutrophil elastase and many other protein components. Beside their role as a host defense mechanism, NETs play a key role in other non-infectious processes including thrombotic disease, vasculitis, wound healing, autoimmunity, metastatic dissemination and cancer associated thrombosis. Previous studies reported indeed that NETs promote thrombosis by providing a scaffold for platelet and red blood cell adhesion and aggregation and by enhancing coagulation [14, 15]. Biomarkers of NETs have been found in thrombi and plasma of both animal models and patients with deep venous thrombosis [15, 16].

Zuo et al. [17] were the first to highlight the role of NETs in the pathogenesis of severe manifestations of COVID-19 patients by reporting that NETs are increased in plasma of COVID-19 patients. These findings were confirmed by at least 3 additional studies [18–20]. Neutrophils of COVID-19 patients are able to release higher levels of NETs and viable SARS-CoV-2 can directly induce NET release from neutrophils of healthy donors. Furthermore, pulmonary autopsies confirmed NET-containing microthrombi with neutrophil-platelet infiltration. Interestingly, we have recently shown that the web-like structure of NETs contains fibronectin, a protein serving as an adhesion substrate for integrin-expressing host or cancer cells [21, 22]. Furthermore, integrins have been reported to contribute also to SARS-CoV-2 docking and cell entry [23]. Since integrin imaging is possible using radiolabeled RGD peptides, it is conceivable that these tracers may be employed to detect lung damage in COVID-19 patients. A recent case report [24], showed that αvβ6 integrin expression can be visualized using 18F-labeled αvβ6-binding peptide in lung lesions 2 months after the acute phase of SARS-CoV-2 infection indicating that integrin imaging may have a role in monitoring the persistence and progression of lung damage in COVID-19 patients.

## Conclusions

In conclusion, dysfunction of immune system and prothrombotic state are the main intersections between COVID-19 and cancer. 18F-FDG PET/CT in cancer patients may provide clues to better understand the pathogenesis of multiorgan damage and severe clinical manifestation of COVID-19. New tracers or revisitation of known radiopharmaceuticals are needed to test specific pathogenetic hypothesis.

## Data Availability

No Data associated in the manuscript.
